# Phytonano silver for cosmetic formulation- synthesis, characterization, and assessment of antimicrobial and antityrosinase potential

**DOI:** 10.1186/s11671-024-04008-6

**Published:** 2024-04-15

**Authors:** Neethu George, D. Gayathri Devi

**Affiliations:** 1 Department of Life Sciences, University of Calicut, Malappuram, Kerala 673635 India; 2 Department of Biochemistry, Pazhassiraja College, Pulpally, Wayanad, Kerala 673579 India

**Keywords:** Tyrosinase, Melanin, Green synthesis, Silver nanoparticle

## Abstract

Novel formulations of silver nanoparticles remain exciting if it is applicable for cosmetic purposes. This study proposes a value-added brand-new nanomaterial for improving skin complexion by inhibiting melanin development. This work aims to develop cost effective, efficient, natural silver nanoparticles phytomediated by aqueous extract of leaf sheath scales of *Cocos nucifera* (Cn-AgNPs) having potential as tyrosinase inhibitors hindering melanin synthesis. The formation of Cn-AgNPs was assessed spectrophotometrically and confirmed by the sharp SPR spectrum at 425 nm. The chemical composition profiling was characterized by X-ray diffraction (XRD) and Fourier Transform Infrared (FTIR) spectroscopy. The morphology was confirmed by Field Emission Scanning Electron Microscopy (FESEM) and the thermal stability was assessed by Thermogravimetric analysis (TGA). Pharmacological application studies supported the materialization of Cn-AgNPs with significant antityrosinase potential and considerably improved antibacterial and antioxidant properties. Cn-AgNPs showed potential antibacterial effects against gram-positive and negative strains, including prominent infectious agents of the skin. Antioxidant capacity was confirmed with an IC_50_ of 57.8 μg/mL by DPPH radical scavenging assay. Furthermore, in vitro melanin content determination was performed using SK-MEL cells. Cell line studies proved that Cn-AgNPs decrease the melanin content of cells. The IC_50_ value obtained was 84.82 μg/mL. Hence Cn-AgNPs is proposed to be acting as a whitening agent through lessening cellular melanin content and as a significant inhibitor of tyrosinase activity. The antioxidant properties and antibacterial effects can contribute to skin rejuvenation and can prevent skin infections as well. This evidence proposes the development of a new nanostructured pharmaceutical and cosmetic formulation from *Cocos nucifera* leaf sheath scales.

## Introduction

The cosmetics business has expanded in recent years, creating more appealing and inventive methods as well as natural goods. As a result, even though natural ingredients have been used for centuries to treat skin conditions, consumers are increasingly demanding natural ingredients in cosmetic products rather than synthetic ones that could have detrimental effects on their health or the environment [1]. This demand is evident in many formulations. Thus, there is a rise in buying of environmentally friendly goods that have added advantages, such as the benefits of plant extracts for skin care [2]. Researchers have given tyrosinase a great deal of thought because of its critical function in the enzymatic browning of food and depigmentation problems in humans. As previously indicated, there is a great deal of promise for organic anti-tyrosinase supplies derived from natural sources including plants and microbes and their powerful chemicals [3]. In dermatology and cosmetology, metal and metal oxide nanoparticles (NPs) are being used more and more, particularly in the prevention and treatment of bacterial and fungal infections, in sun protection, and in preparations that lessen the visibility of scars by speeding up skin cell repair. Nanoformulations can also be utilised for dermatological procedures and skin care to enhance patients' quality of life. By providing fast-acting, safe, ecologically friendly and effective product formulations, nanodermatology and nanocosmetology help reduce the negative effects of the products that have already been utilised [4]. Metal and metal oxide nanoparticles have a wide range of dermato-cosmetic uses, such as providing targeted, controlled medication release and protecting the skin from UV and microbes [5]. Green synthesised nanoparticles resorting to various plant extracts show high potential towards its clinical translation to dermatopharmaceutical and cosmetic applications [6].

Nanomaterials synthesised via green synthetic route develop substantial interest in research fraternity since its smaller size facilitates favourable physical, chemical, optical, magnetic and biological properties [7–11]. Establishing a good biosynthetic pathway for a nanoparticle with emphasise on human health is worthwhile. The green technique is preferred since it has a lower impact on toxicity, fewer biological risks, and uses less energy [12]. The antibacterial [13], antiviral [14], antifungal [15], anti-inflammatory [16], antiseptic [17] and anticancer[18] properties of green synthesised nanoparticles are well established. Among these silver nanoparticles has already been applied in various fields i.e. electronics, optics, catalysis, food, health and environment [19]. In packaging [20] textile coatings [21], wound dressings [22, 23], cleaning products [24], cosmetics [25], pharmaceutical gel [26], and against skin diseases[25] silver nanoparticles are often employed.

*Cocos nucifera*, a pantropical plant is one of the major perennial oil crops commonly known as the coconut palm. It serves as a basis for food production in developing countries and described as one of the natures gift to man [27]. Different parts of the Coconut palm are well established in the synthesis of nanoparticles which are reported to be applicable as antimicrobial agents [28, 29], antioxidants [30], drug delivery systems [31], anticancer and photocatalytic agents [32]. *Cocos nucifera* mediated silver nanoparticles are established against staphylococcal infections [33], dengue vector [34] and possess larvicidal [35] activities.

One of the specialised enzymes in the melanosome, tyrosinase, is a rate-limiting enzyme in the melanogenesis pathway and is found in humans, plants, bacteria, and fungi. It facilitates the hydroxylation of monophenols to produce o-diphenol (monophenolase activity), o-quinone (diphenolase activity), and subsequently melanin [36]. Pigmentation, including melasma, age spots, freckles, and melanoma, is caused by accumulation of melanin [37]. As a result, there is a lot of interest in the quest for potent tyrosinase inhibitors in cosmetic industry. Tyrosinase inhibitors made using a green chemical method are reported to have fewer negative effects than synthetic inhibitors [38]. The discovery of natural anti-tyrosinase agents that are both affordable and potentially successful for whitening severely pigmented lesions is urgently needed in the cosmetics sector [39]. The skin is the most affected organs by the various detrimental environmental variables that lead to its senescence and ageing since it is constantly exposed to the outside world. Overproduction of reactive oxygen species (ROS) in the skin causes intrinsic ageing of the skin and eventually leads to wrinkle development. The primary process in the pathophysiology of wrinkle development is the cleavage of the fibrous proteins that make up the skin's extracellular matrix (ECM), such as collagen, elastin, and hyaluronic acid, by the ROS-induced enzymes collagenase, elastase, and hyaluronidase, respectively [40]. One of the most effective ways to prevent skin ageing is to stop the ROS production which leads to the breakdown of the extracellular matrix (ECM) of skin and minimise melanin synthesis and pigmentation. A number of plant extracts and secondary metabolites show promise as anti-aging options and have the added advantage of possessing strong antioxidant properties [41]. Researchers have been working on elucidation of novel materials by green approach for cosmetic purpose in order to reduce biohazards. Furthermore, the synthesis of silver nanoparticles from plant sources are more advantageous than other synthetic methods. Silver nanoparticles are preferred in cosmetic formulations owing to their antifungal and antibacterial properties [42]. Synthesis of tyrosinase inhibitors through green chemistry approach are reported to have lower side effects comparative to synthetic inhibitors [43]. There is a great need in cosmetic industry for the development of cost effective natural and prospective anti tyrosinase for bleaching of extremely pigmented lesions.

In this work we designed a simple and rapid methodology for synthesis of silver nanoparticles using *Cocos nucifera* leaf sheath scales as reducing agent. Anatomically *C. nucifera* leaf sheath scale is the cell debris of the actively dividing meristematic tissue, which forms the fibrous nature [44]. Although the wound healing and scar bleaching properties of the leaf sheath scales of *Cocos nucifera* are exploited in folk medicine, no attempts have been made so far to produce a value added cosmetic product from this plant part. Hence, we describe a simple, eco-friendly and cost effective process for the green synthesis of AgNPs utilising *C. nucifera* leaf sheath scale extract (Cn-AgNPs). However, notwithstanding the surplus number of papers, to date no information about the green synthesised silver nano formulation from *C. nucifera* leaf sheath scale with the intention of employing as a skin rejuvenator by virtue of its antityrosinase, antimelanogenic, antimicrobial and antioxidant properties is available. Based on our findings, we propose that Cn-AgNPs could confer tolerance to oxidative stress and have great potential against melanogenesis and showing antibacterial properties as well.

## Materials and methods

### Preparation of plant extract and synthesis of silver nanoparticles

**Scheme 1:**
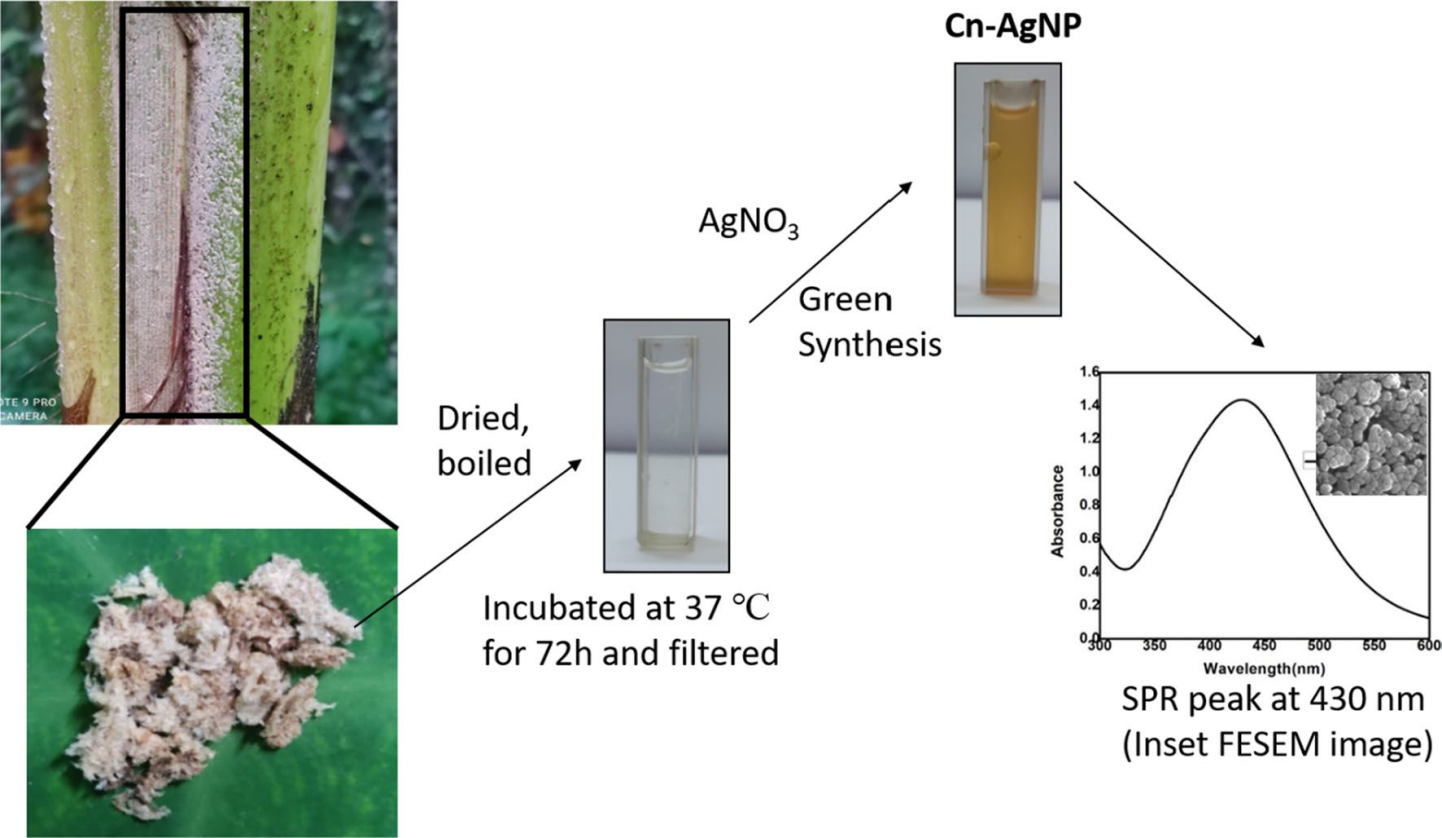
Preparation of plant extract and synthesis of silver nanoparticles

Coconut leaf sheath scales were collected from the outskirts of Vadakara, Kozhikode district Kerala, India in the month of April and identified by the Department of Botany, University of Calicut. The plant specimen was deposited in the Calicut University herbarium CALI (Accession number: 7186). The collected Coconut Leaf Sheath Scales were shade dried and boiled in distilled water for 45 min. This solution was kept in shaker for 72 h at 37 °C. The mixture was cooled and filtered through Whatman filter paper. The extract was collected and utilized for synthesis of silver nanoparticles as per earlier protocol [45]. To 90 mL of 1 mM Silver nitrate solution 10 mL of *C. nucifera* leaf sheath scales aqueous extract (Cn extract) was added and the mixture was boiled which is diagrammatically represented in Scheme 1. The synthesis of silver nanoparticle by reduction of Ag^+^ to Ag^0^ was observed from the color change of the solution.

### Characterisation of biogenic silver nanoparticles

Visual inspection of the color shift was the indicator of the formation of AgNPs. Spectrophotometric detection of the absorption peak using a UV–Vis spectrophotometer (Shimadzu UV–Visible 1800 spectrophotometer) was used to validate the generation of silver nanoparticles via green route utilizing *C. nucifera* leaf sheath scale extract (Cn-AgNPs). A number of factors were looked at in order to get the best possible experimental circumstances, including the incubation period, temperature, precursor concentration, and plant extract concentration. Shelf life and pH stability of Cn-AgNPs were also assessed spectrophotometrically. The response of functional groups present in the plant extract possibly involved in the behavior and properties of the synthesized Cn-AgNPs and were examined using FT-IR spectrometer (ATR FTIR Perkin–Elmer). Using a diffractometer to record the X-ray diffraction (XRD) pattern, it was possible to analyse the crystalline nature and phase definition of the structure of Cn-AgNPs. Malvern PANalytical Xpert3 Powder diffractometer was used to record the X-ray diffraction pattern, and the data obtained was studied to evaluate the crystalline nature and phase definition of the structure of Cn-AgNPs and also for the theoretical calculation of the size of the particle. The X-ray generator was operated at a voltage of 45 kV and a current of 30 mA, wherein the sample was subjected to CuKα radiations (λ = 1.54059 Å) at 2θ angle. Further, the images obtained were compared with the Joint Committee on Powder Diffraction Standards (JCPDS) library to account for the crystalline structure of the particle [46]. The surface morphology of Cn-AgNPs was analysed using FE-SEM. Freeze dried Cn-AgNPs were observed under FESEM (JSM-6700F, Jeol Ltd, Tokyo, Japan). The thermal characteristics of the freeze-dried nanocrystals was examined by TGA measurements in order to investigate the specifics of the heat-assisted disintegration of Cn-AgNPs. TGA experiments performed were utilized to measure weight loss and thermal stability of the bio-reduced nanoparticles. It was carried out on a thermal analyzer (Perkin Elmer STA 8000) within the temperature range of 30–800 °C at a heating rate of 10 °C per min under the inert atmosphere of argon [47, 48]. To confirm the average size of the synthesized silver nanoparticle and to ensure storage stability DLS measurements were done [49].

### Anti-tyrosinase effects

Dopachrome method described by Abdhillahi et al. [50] was employed with slight modifications. Tyrosinase inhibition can be measured by spectrophotometric analysis using L-Dopa as substrate. The reaction mixture (1.0 mL final volume) contained 1 mM L- Dopa, 50 mM Sodium Phosphate buffer and 200 U Tyrosinase enzyme. 50 µM Kojic acid was used as positive control. Samples were incubated at 30 °C for 20 min. The dopachrome developed was monitored at 475 nm by using Shimadzu UV–Visible 1800 spectrophotometer. The relative Tyrosinase activity can be calculated by using the following equation:

$$Tyrosinase\; inhibitory\; activity=\frac{\left(A-B\right)-(C-D)}{(A-B)}\times 100$$

where A is absorbance of reference solution after incubation, B is absorbance of reference solution before incubation, C is absorbance of sample solution after incubation, and D is absorbance of sample solution before incubation. The absorbance of reaction was taken after the deduction of their respective blanks.

### Melanin content determination on SK-MEL cell lines

The melanin content was measured following the method described by Bayrakçeken Güven et al. [51] with slight modifications. SK-MEL cells are an exceptional source of melanin [52] SK-MEL cells (5 × 10^4^ cells) were seeded overnight in 6 well plates. Subsequently, the cells were treated with different concentrations (6.25- 100 μg/mL) of the test compounds. The cells were cultured for 96 h; after that, the cells were harvested and the cell pellets prepared. The cell pellets were dissolved in 200 μL of 1N NaOH containing 10% DMSO for 1 h at 80 °C. After centrifugation at 12,000 g for 10 min at 25 °C, the supernatant was collected. The cell lysate (100 μL) was pipetted into a 96-well microplate, and the absorbance at 490 nm was determined using a Microplate Reader. The absorbance values were normalized to the protein content in the cell lysates. The protein concentration in the cells was estimated by the Bradford method [53].

### Antioxidant activity

DPPH radical Scavenging assay was used to investigate antioxidant activity of Cn-AgNPs [54]. Different concentrations (25, 50, 75, and 100 µg/mL) of green synthesised silver nanoparticles (Cn-AgNPs), *C. nucifera* leaf sheath scale extract (Cn-extract) were exposed to 1 mL of DPPH (0.1 mM in ethanol) solution. The mixture was kept in room temperature for 10 min with vigorous shaking. Absorbance at 517 nm was measured using a UV/visible spectrophotometer. Ascorbic acid (AA) was used as a reference compound. A mixture of Ascorbic acid and Cn-AgNPs was also subjected to analysis. The sample has a higher level of free radical activity when the absorbance is lower. The percent DPPH radical scavenging activity was calculated by using the following equation.

$$\% \;of\; inhibition=\frac{Absorbtion\; of\; control-Absorbtion\; of \;test}{Absorbtion \;of \;control}\times 100$$

### Antibacterial activity

#### Agar well diffusion method

Agar well diffusion method was employed to assess the antibacterial activity of Cn-AgNPs [55]. Most common infection causing Gram-positive *Staphylococcus aureus* (ATCC 25923) and Gram-negative *Escherichia coli* (ATCC 25922) were tested to evaluate the antibacterial efficiency of Cn-AgNPs synthesised via green approach. The studied organisms were cultured in LB broth overnight before being transferred to an LB agar plate. The bacterial cultures were treated with different concentrations of Cn-AgNPs (25, 50, 75, and 100 µg/mL) and the plates were incubated for 24 h at 30 °C. The zone of inhibition of each well were measured and the readings were recorded. The sterile cork borer (8 mm) made wells were filled with samples. The control groups comprised of chloramphenicol, plant extracts, AgNO_**3**_ and purified water separately. The inhibitory zones were measured with digital callipers and compared with the inhibitory zones of the control groups.

#### Resazurin based broth microdilution assay

The minimum inhibitory concentration of synthesised Cn-AgNPs was analysed by broth microdilution assay[56], performed using the Resazurin dye metabolization technique in a 96-well polystyrene flat bottomed microtiter plate. The bacterial strains were seeded on to 96-well plate along with different concentrations of Cn-AgNPs ranging from (100 µg/mL to 0.39 µg/mL) and the plates were incubated for 24 h at 30 °C. Rezasurin dye were used as an indicator of growth. Serial dilutions were made from the first well by adding 100 µL of bacterial culture and 100 µL of Cn-AgNPs. 30 µL of resazurin indicator was added after 18 h of incubation of the bacterial suspension along with Cn-AgNPs at 37 °C. We could see the indicator's colour shift from purple to pink as a sign of microbial growth after 2 h of incubation in the dark. The MIC value was determined to be the lowest concentration of Cn-AgNPs at which discolouration occurred. The first four wells' contents were plated on an LB plate for analysis of the minimum bactericidal concentration (MBC) and incubated for 24 h. MBC was identified as the lowest broth dilution of an antibiotic that inhibits an organism's ability to grow on an agar plate.

### Statistical analysis

All data were presented as mean ± standard error. All experiments were independently performed three times. The mean values of the treatment groups were compared with untreated groups using Students t test. Statistical significance was assigned at *p* < 0.05.

## Results and discussion

### Visual observation

**Fig. 1:**
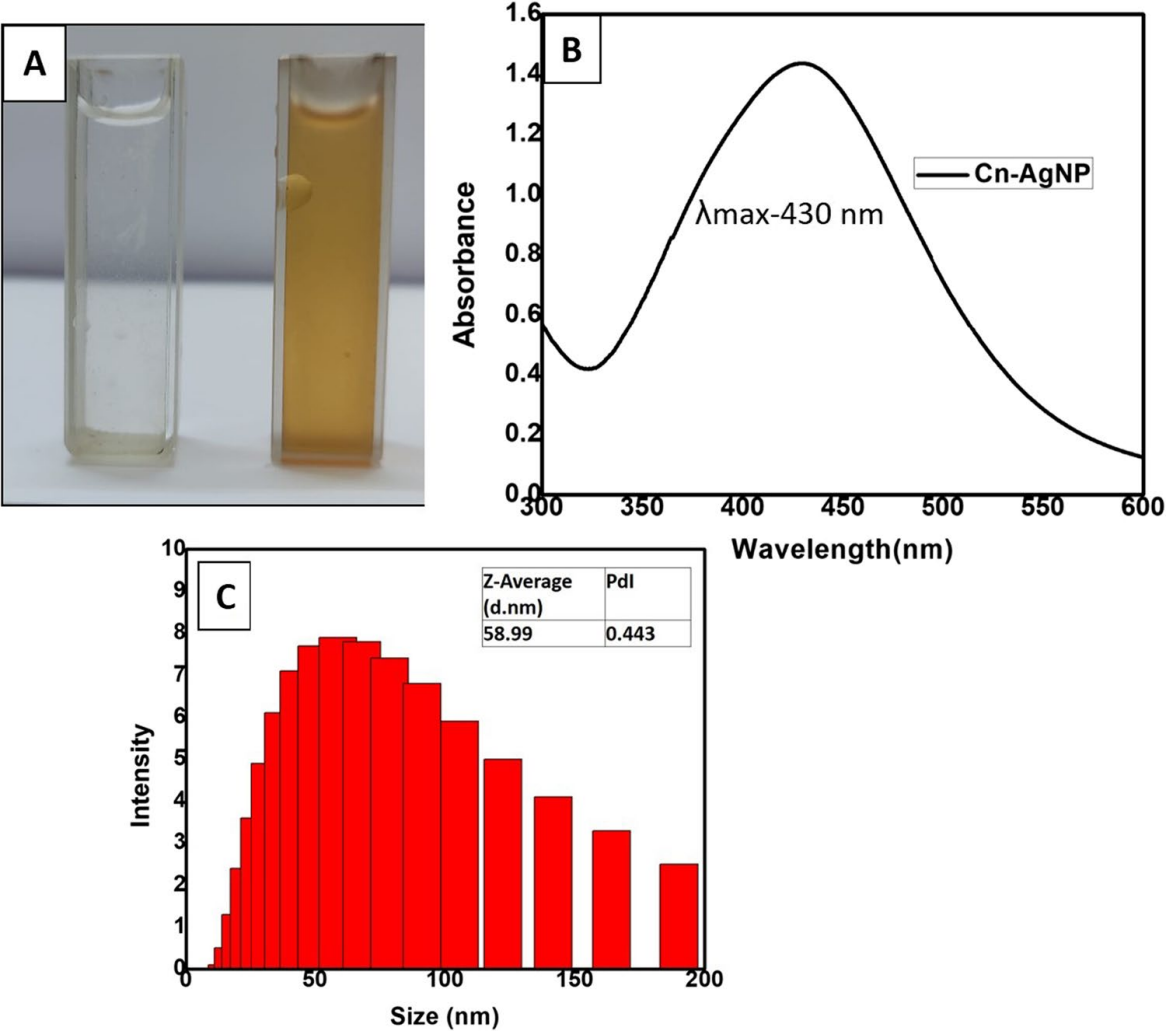
**A** Visual observation of the color of aqueous *extract of C. nucifera leaf sheath scale* and change in color of the solution when aqueous *C. nucifera leaf sheath scale* added to 1 × 10^–3^ M AgNO_3_. **B** UV–Visible absorbance spectra of Cn-AgNPs optimised under the conditions of 60 ℃, 30 min and treated with 1 mM AgNO_3_ and 1000 µL of Cn extract. **C** DLS data showing the size distribution of Cn-AgNPs

Phytoreduced silver nanoparticles using leaf sheath scales of *C. nucifera* were created using an eco-friendly process. The colour of the silver nitrate solution changes when plant extract is added, going from light yellow to yellowish-brown to reddish-brown as seen in Fig. 1A. The plant moiety in *C. nucifera* leaf sheath scales which includes ketones, amides, esters, unsaturated nitrogen compounds, polyphenols, sulphur compounds, halogen compounds, some unknown phytochemical radicals, fatty acids and cholesterol [57] is what reduces and stabilises the nanoparticles. The capacity of plant extracts biomolecules to donate electrons may help to explain AgNPs formation which led to the conversion of Ag+ ions into Ag metal. Additionally, the functional groups of the bioactive metals may contribute to its stability [58]. The colour shift observed during nanoparticle creation is due to the AgNPs' surface plasmon resonance (SPR) vibration being excited.

### Characterisation of nanoparticles

#### UV–visible spectroscopy

**Fig. 2:**
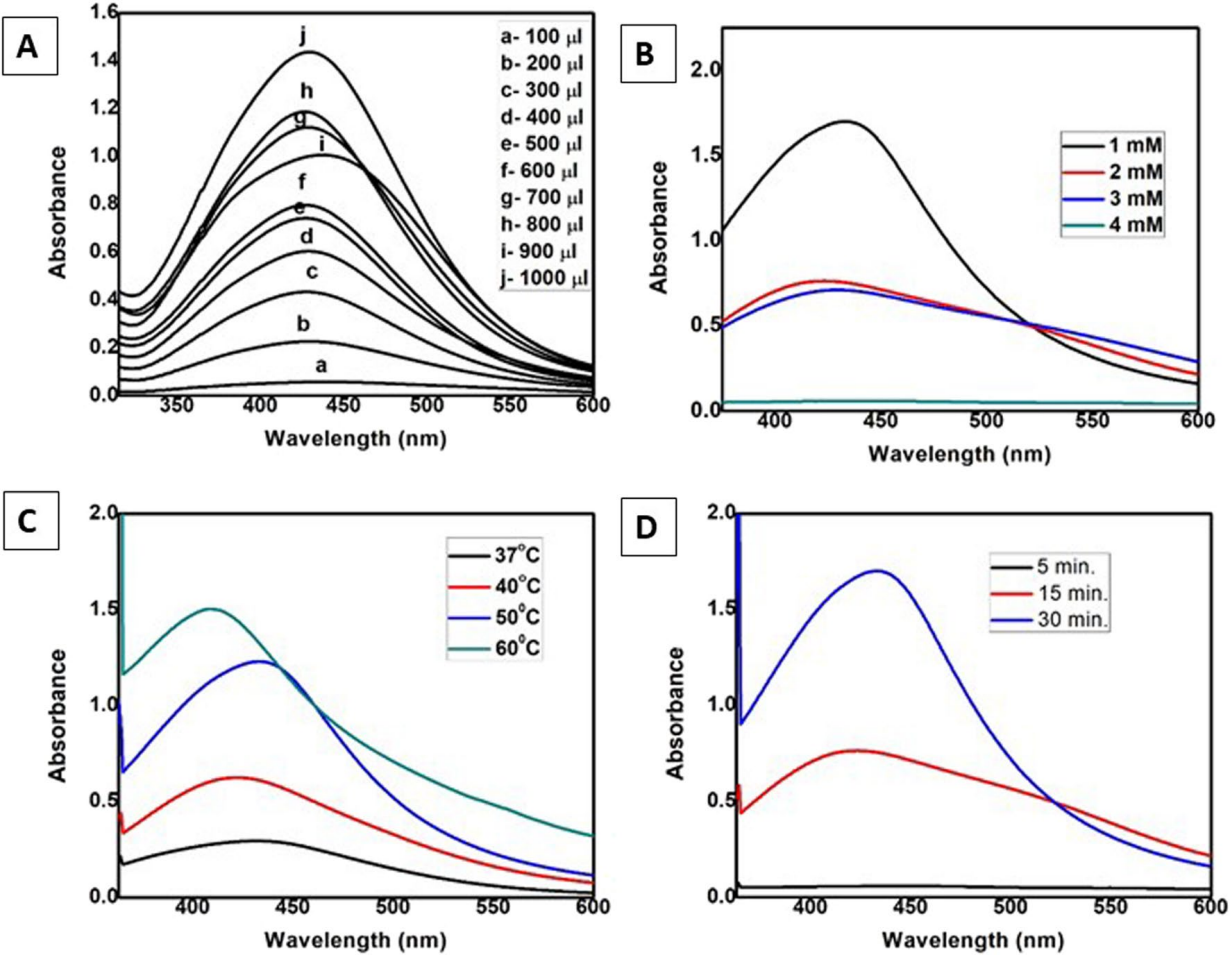
**A** UV/Vis spectral readings of Cn-AgNPs synthesized by treating 1 × 10^–3^ M AgNO_3_ with different volumes of 10% *C. nucifera* leaf sheath scale extract. **B** Absorption kinetics of Cn-AgNPs with varying concentrations of AgNO_3_. **C** Absorption spectrum of silver nanoparticles treated with *C. nucifera* leaf sheath scale (10%) with AgNO_3_ (1 mM) at different temperatures. **D** Spectral readings of Cn-AgNPs observed at different reaction times when treating 1 mM AgNO_3_ with aqueous *C. nucifera* leaf sheath scale extract

The formation and stability of the synthesised biogenic Cn-AgNPs were first confirmed by UV–Visible spectroscopy. The characteristic absorbance, SPR range from 410 to 430 nm confirmed the reaction progress and completion of reaction. The stability of the green synthesised nanoparticles was validated by measuring absorbance at various times. Figure 1B shows the SPR peaks of Cn-AgNPs obtained at 430 nm under optimised conditions of 60 ℃, when treated with 1 mM AgNO_3_ and 1000 µL of Cn extract for 30 min. Figure 2A shows the increasing absorption spectrum on increasing the concentration of extract. A sharp spectrum of 430 nm was obtained for the silver nanoparticles synthesized using 1000 µL of 10% plant extract. Strong Plasmon bands around 430 nm represent the reduction of Ag^+^ ions to Ag^0^ with this minimum concentration of *C. nucifera* leaf sheath scale extract. Decrease in the peak height of the surface plasmon band was observed (Fig. 2B) when treating 1000 µL of 10% plant extract with increasing silver concentration. It may be noticed that 4 mM AgNO_3_ shows no characteristic peak at 430 nm. It may be due to the insufficient amount of phytoconstituents available for the reduction of higher concentrations of AgNO_3_. Metallic nanoparticle experiences the color change depending on the size of the nanospheres. The transverse absorption peak may shift right if the radius of the sphere increases [59]. Slight red shift in the plasmon band was observed with the increase in temperature. But it returned back to the initial wavelength when the temperature was further increased and reached up to 60 °C (Fig. 2C). Figure 2D shows a positive correlation between the nanoparticle concentration and period of incubation. After 30 min of incubation, no further growth was observed. Hence 30 min of incubation was found to be optimal incubation time for the green synthesis of Cn-AgNPs.

**Fig. 3:**
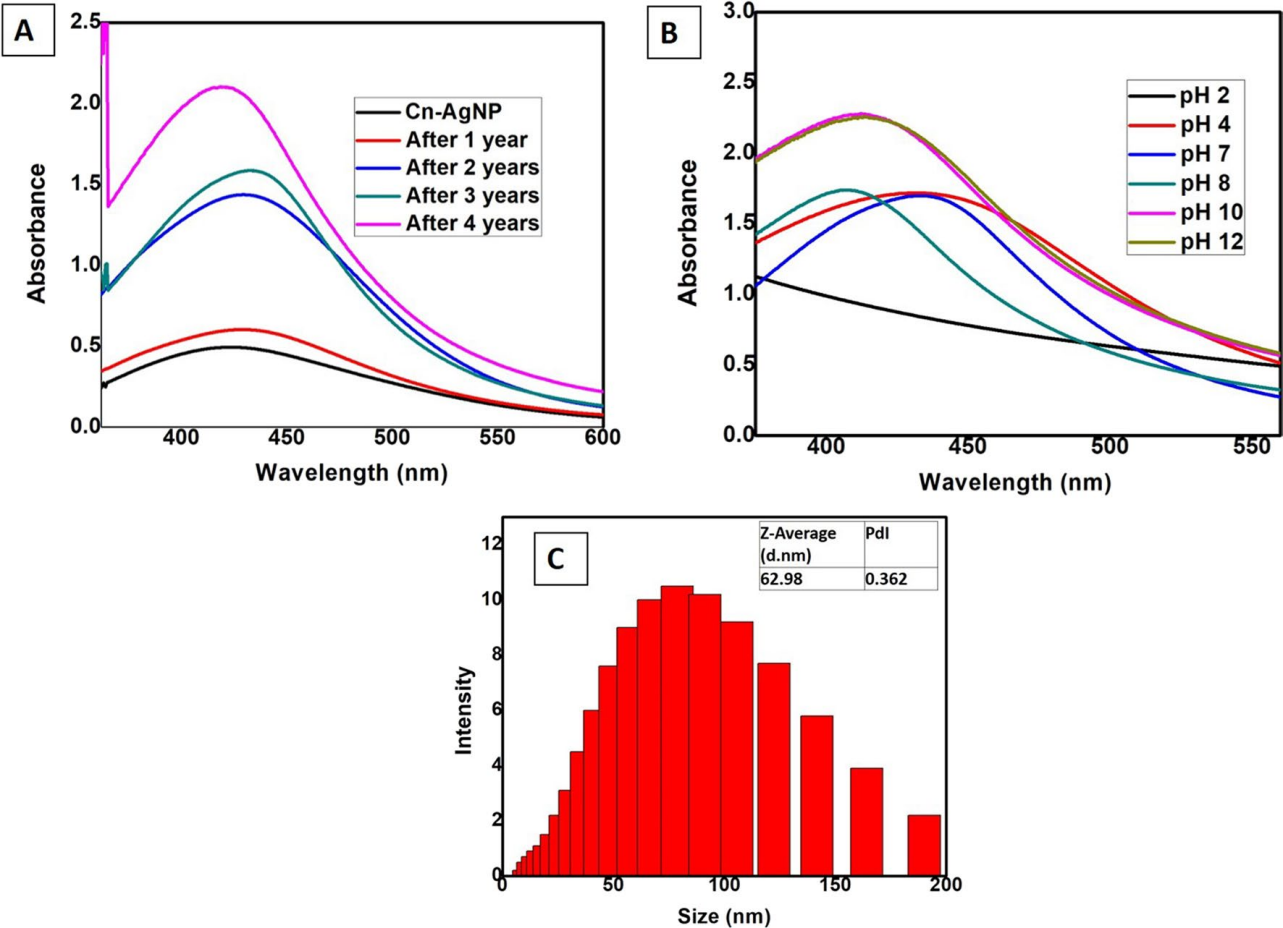
**A** UV–Visible spectra showing the storage stability of synthesized Cn-AgNPs. **B** Absorption spectra exhibiting the pH stability. **C** DLS data confirming the stability of Cn-AgNPs stored for 5 years

Under acidic and alkaline circumstances, it was observed that the nanoparticles were maintaining a typical absorption maximum in the range of 400–450 nm (Fig. 3B). It is reported that, severe pH variations can modify the shape and structure of nanoparticles by warping the structures of biomolecules, which can impact their ability to stabilise [60]. In the current investigation, Cn-AgNPs demonstrated remarkable stability throughout a pH range of 2 to 12, indicating their potential in using both acidic and alkaline settings. An additional way to verify the viability of a nanomaterial for various applications is to assess the shelf-life [61]. The absorption spectrum over the years (Fig. 3A) clearly shows that the Cn-AgNPs maintained the absorption maximum at a wavelength between 400 and 450 nm at room temperature. The electrochemical potential difference causes a more stable interaction between phytoconstituents and silver ions [62]. Storage stability was further confirmed by DLS (Fig. 3C), which shows an average hydrodynamic diameter of 62.98 ± 1.65 nm in par with the size of the freshly prepared nanoparticles. Considerable shelf-life of the synthesised Cn-AgNPs is promising, and its ability to survive in the room temperature is more advantageous.

#### DLS analysis

The nanoparticle size distribution was investigated by Dynamic Light Scattering. The DLS graph of Cn-AgNPs shows that the average hydrodynamic diameter of the population of nanoparticles is 58.88 ± 5.7 nm (Fig. 1C) which is in agreement with the size displayed in SEM images and the theoretical value obtained from Debye–Scherrer formulae. Upturn in the numerical value of DLS data can be attributed to the hydrodynamic diameter that cover the electrical double layer of solvent molecules.

#### FESEM

**Fig. 4:**
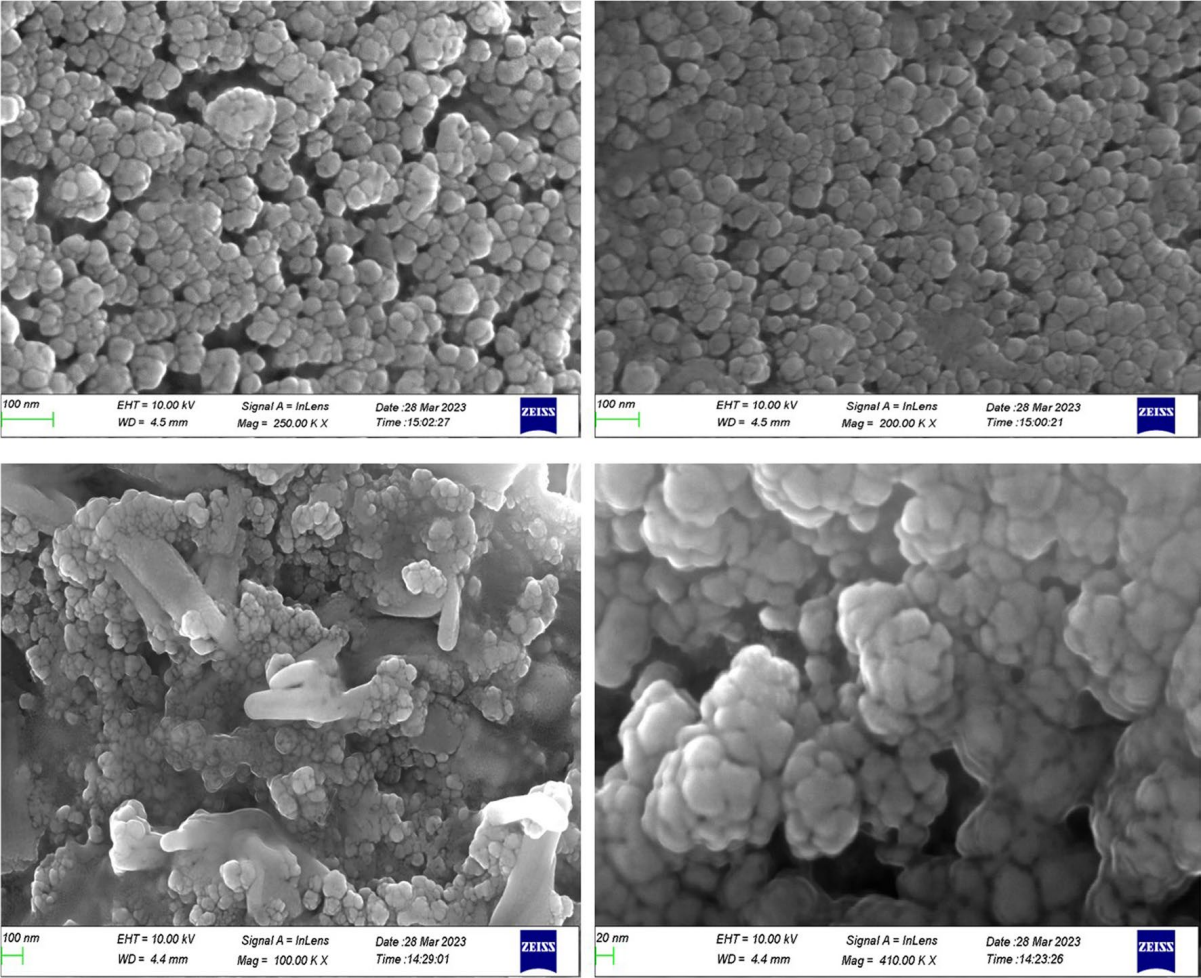
Scanning Electron Micrographs of Cn-AgNPs at different magnifications

The surface morphology of the synthesised nanoparticles was analysed by FESEM technique. It clearly showed spherical shaped nanoparticles. The size of the particles is seen to be uniform and of 28 nm in diameter. Most of the particles were aggregated and the images confirm the formation of biomolecules capped with biomoieties (Fig. 4).

#### Fourier-transform infrared spectroscopy

**Fig. 5:**
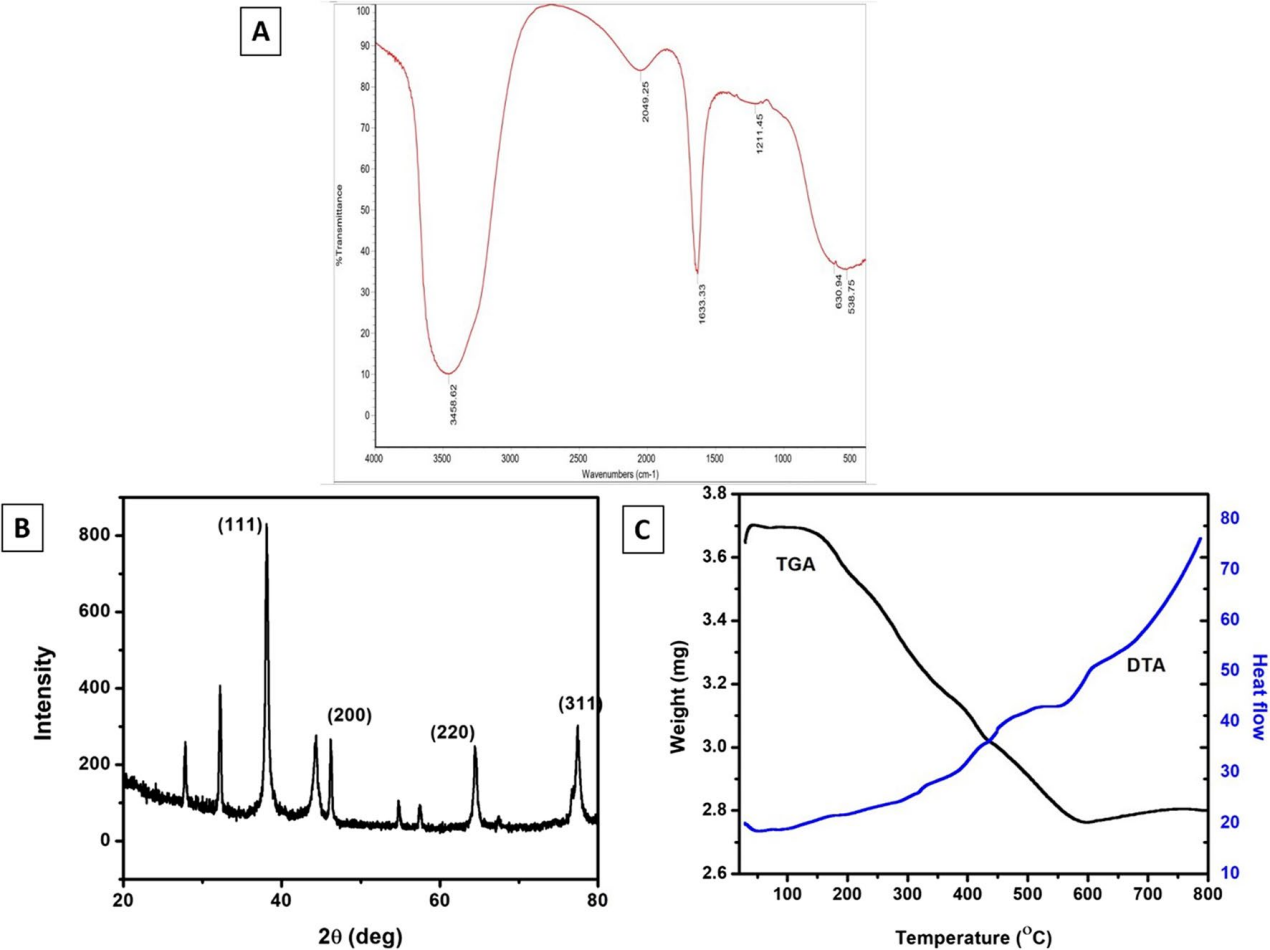
**A** FT-IR spectral details of silver nanoparticles bioinspired by aqueous *extract of C. nucifera* leaf sheath scale*.* **B** XRD pattern of Cn-AgNPs fabricated by treating 1 × 10^–3^ mM AgNO_3_ with aqueous *extract of C. nucifera* leaf sheath scale*.* **C** Thermogram of Cn-AgNPs

The FT-IR studies confirmed the functional groups present on the surface of Cn-AgNPs. Absorption spectrum shows an absorbance band in the region 538.75–3458.62 cm^−1^ (Fig. 5A). The bands absorbed in this region were 3458.62, 2049.25, 1633.33, 1211.45, 630.94 and 538.75 cm^−1^. 3458.62 cm^−1^ typically corresponds to the stretching vibration of O–H bonds, indicating the presence of hydroxyl groups which signifies the presence of functional groups like alcohol or phenol groups. This may indicate the presence of carbohydrate and proteins. Compounds such as alkynes or nitriles typically exhibit absorption in the region of 2049.25 cm^−1^ which is located in an area where triple bond stretching vibrations are known to occur. It could be related to the stretching vibrations of triple bonds between carbon and nitrogen (C≡N) or between carbon atoms (C≡C). Presence of functionalities like ketones, aldehydes, carboxylic acids, esters, and amides was confirmed by the band at 1633.33 cm^−1^ corresponding to the stretching vibrations in carbonyl groups. Generally, the peak observed at 1211.45 cm^−1^ represents the stretching vibration of C–O bonds, particularly in alcohols, ethers, and carboxylic acids [63]. It is also related to the stretching vibration of the C–N bond in amines. 630.94 cm^−1^ and 538.75 cm^−1^ are located in the fingerprint area of  FTIR spectrum. It may result from different functional group bending vibrations, such as C–H bonds in aromatics, methyl groups, or alkanes. It confirms our hypothesis that the creation and stabilization of nanoparticles are mediated by a variety of phytochemical elements found in leaf extract, including alkaloids, amino acids, flavonoids, saponins, steroids, glycosides, carbohydrates, and tannins[64]. The phytoconstituents found in the aqueous extract of *C. nucifera* leaf sheath scales play a key role in modulating the size of the nanoparticles. It serves as capping as well as reducing agent. The FTIR spectra make it abundantly evident that the phytocomponents alone are in charge of the production and stability of Cn-AgNPs.

#### X-ray diffraction studies

XRD analysis was used to assess the phase formation, purity, and crystallinity of the synthesised nanoparticles. The nanocrystalline nature of the particle can be confirmed by the XRD patterns and the numbers of Bragg reflections can be linked to the face-centered cubic structure of silver. The lattice microstrain and crystallite size are inversely correlated with the diffraction peak broadening. The size distribution, strain, and defects in the nanocrystals can all be explained by the broadening and widening of the diffraction lines [65]. The cubic silver's (JCPDS 00-004-0783) (111), (200), (220), (311), and (222) reflections are indexed in the XRD patterns (Fig. 5B). The crystalline nature of the AgNPs synthesised with the aid of Cn extract is evident from the presence of these structural peaks in the XRD pattern. According to previous scientific findings, the resulting diffraction pattern is consistent with that of silver [66]. Using the Debye Scherrer equation, the crystallite size of the nanoparticles was determined and tabulated in Table 1 [67].

$$D=\frac{K\lambda }{\beta Cos\theta }$$

where D is the AgNPs' crystalline size, "λ" is the X-ray source's wavelength (λ = 1.540598 A0) used in the XRD pattern, "β" is the full width half maximum of the conspicuous diffraction peak in radians, "K" is the Scherrer constant (0.9), and "θ" is the Braggs angle.

**Table 1:** Calculation of the size of AgNPs by using Debye–Scherrer’s equation theoretically, the average size of the nanoparticles was calculated to be 21.94 nm

2θ	FWHM	β	θ	Cos θ	D (nm)
38.1223	0.4455	0.007775	19.06115	0.977697	18.24
44.3048	0.4475	0.00781	22.1524	0.98703	17.98
64.4411	0.4566	0.007969	32.22055	0.693383	25.09
77.3897	0.4652	0.008119	38.69485	0.543884	31.39
81.5305	0.4686	0.008179	40.76525	0.99715	17

#### Thermal decomposition profile

The lyophilized green nanoparticles were subjected to TGA analysis in order to assess the thermal stability. The weight loss as well as the level of purity of the compound was calculated by estimating the amount of organic moieties capped on the surface of Cn-AgNPs. Figure 5C depicts the thermogram of Cn-AgNPs synthesized with 1 × 10^–3^ M AgNO_3_. Weight loss of 1% to 4% was seen during the first heating stage from 30 to 100 °C, which may have been caused by the sample's moisture content or physisorbed water evaporating. Throughout the second phase, the rate of weight loss remained steady up to 600 °C. A 25% weight loss in volume occurs up to 600 °C due to the breakdown of bioorganic compounds on the surface of silver nanocrystals, which may act as a capping or stabilizing agent after the reduction process. In phase three, Cn-AgNPs began to stabilize. After 600 °C, a slight rise in weight was observed, which is because of the buoyancy caused by the density of air. The bioactive molecules on the surface of Cn-AgNPs are assumed to have been destroyed at higher temperatures, leaving a residual mass of 75% at 600 °C. The remarkable thermal stability of Cn-AgNPs is attributed to the inorganic metal present in the nanocrystals and still have roughly 75% of their weight in terms of purity and stability, based on TGA. There is no decrease in the weight of silver over 600 °C [68].

### Tyrosinase assay

**Fig. 6:**
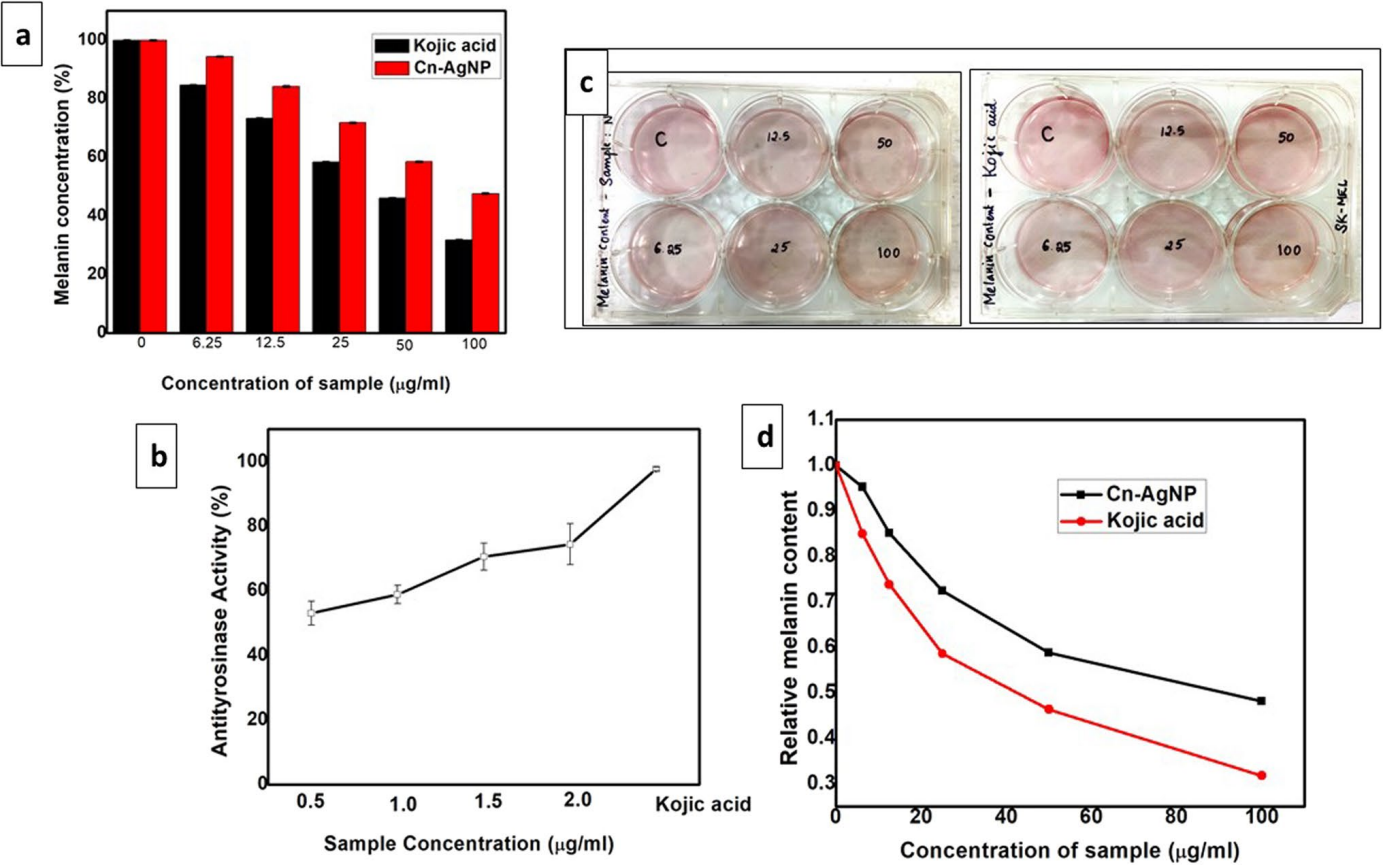
**a** Determination of melanin content in SK-MEL cell lines on treatment with Cn-AgNPs and Kojic acid. **b** Inhibitory potential of Cn-AgNPs against tyrosinase. **c** Image showing SK-MEL cell line on treatment with Cn-AgNPS and Kojic acid. **d** Relative melanin content in presence of Cn-AgNPs and Kojic acid

Aging and skin cancers are associated with DNA damage and melanin synthesis. Chemical mutagens and UV radiations are known to have causative agents of these damages. Environmental contaminants also cause hyperpigmentation [69]. We performed preliminary screening using mushroom tyrosinase as a representative enzyme. The cosmetics industry is interested in finding natural tyrosinase inhibitors since they are thought to have less adverse consequences. Most skin-whitening products target tyrosinase in cells, yet some, like arbutin, may be hazardous and hydroquinone was once widely employed in cosmetic formulations but was discontinued due to unfavourable side effects [70]. Analysis by Dopachrome method showed significant inhibition potential of Cn-AgNPs against mushroom tyrosinase (Fig. 6b) while Kojic acid serves as positive control. Inhibiting this multi copper enzyme considered as a pragmatic approach for the combating melanogenesis. Tyrosinases catalyses the hydroxylation of tyrosine to l-tyrosine to 3,4, dihydroxy phenylalanine (L- DOPA) followed by the oxidation of L-DOPA to dopaquinone [71]. Extract and AgNO_**3**_ alone shows comparatively less inhibition on tyrosinase. But some of the bioactive components of the extract may responsible for the tyrosinase inhibition potential which may be bound with the synthesised Cn-AgNPs. Reports suggest evidences that hydroxyl groups of phenolic compounds or flavonoids of the extract can chelate copper ions in the mushroom tyrosinase, which may leads to the enzyme inhibition [72]. Some green synthesied AuNPs of potential antityrosinase effects but noncytotoxic to cells even at 100% melanin inhibitory concentration are already reported [73] whilst AgNPs synthesised from *Eucommia ulmoides* with ability to suppress both mushroom tyrosinase and A375 cells are also reported [74].

### Anti-melanogenesis activity

To assess the effects of Cn-AgNPs on melanogenesis we measured intracellular melanin content after treatment with Cn-AgNPs in par with kojic acid. Figure 6a shows the dose-dependent reduction in the intracellular melanin content by Cn-AgNPs in SK-MEL cells (5 × 10^4^ cells) after 96-h treatment with Cn-AgNPs. Cn-AgNPs shows comparative inhibition on those treated with same concentration of kojic acid. Figure 6c shows the SK-MEL cell lines on treatment with Cn-AgNPs and kojic acid. The maximum inhibition efficacy was elicited by 100 μg/mL. The IC_50_ value obtained was 84.82 μg/mL. The relative melanin content (Calculated as the ratio between melanin content and protein content in percentage) showed (Fig. 6d) a direct proportionality of reduction with the enhancement in Cn-AgNP concentration. In a previous report, myco synthesized AgNPs ahows significant anti melanogenic activity against human skin melanoma SK-MEL cells which is attributable to our results [75]. *p*-coumaric acid was also identified as an antimelanogenesis agent which was an important tyrosinase inhibitor due its structural similarity with tyrosine [73]. The development of metallic nanoparticles with antimelanogetic effect have been investigated in vitro and in vivo for skin cancer therapy [76]. These observations are an indication that the sample can be used as an agent that can inhibit melanin production and hence can be developed as a cosmetic agent.

### Antioxidant studies

**Fig. 7:**
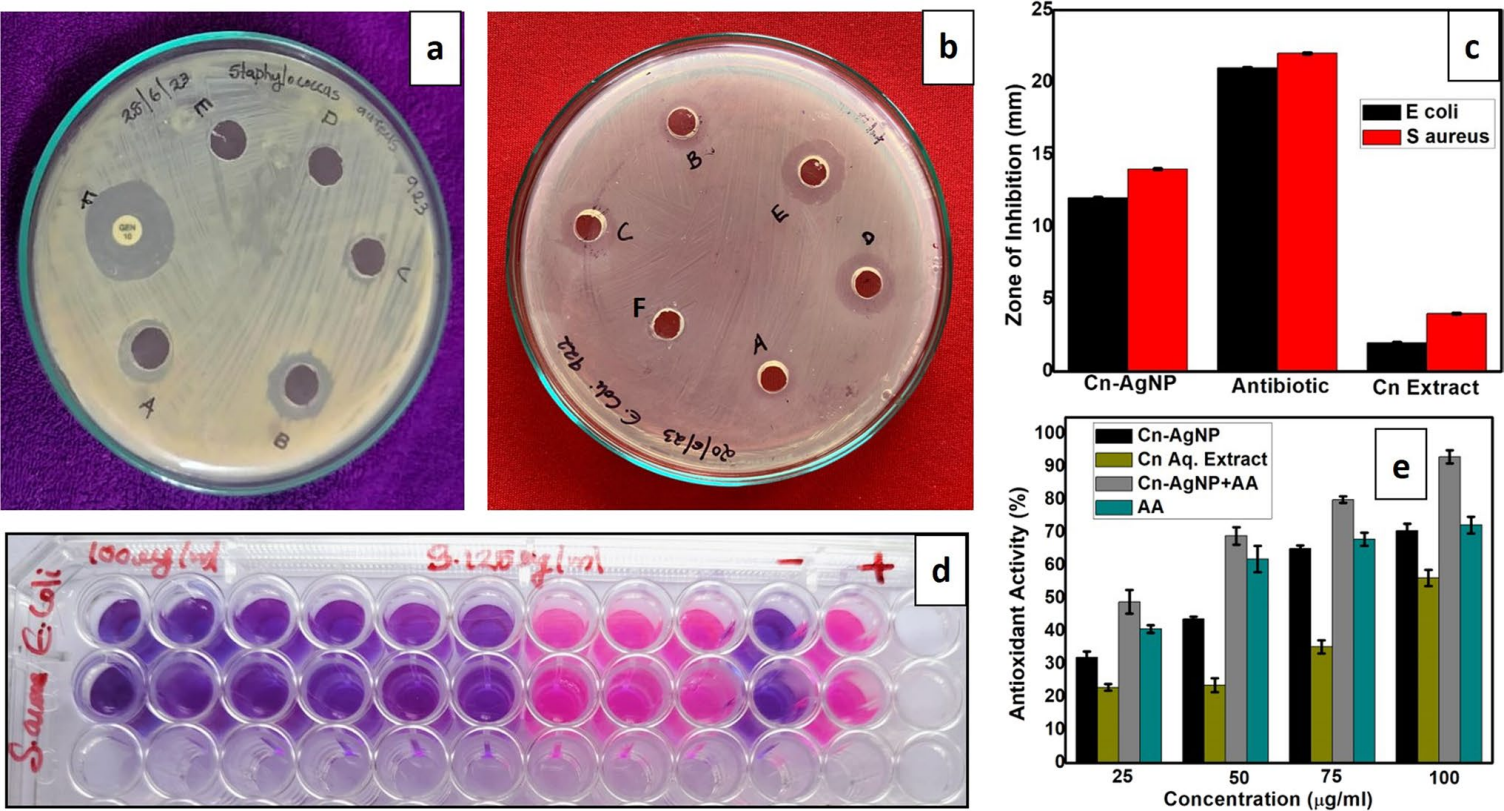
**a** Antibacterial activity of Cn-AgNPs against *S. aureus* when treated with Cn-AgNPs of (A) 75 µg/mL, (B) 100 µg/mL, (C) 50 µg/mL, (D) distilled water, (E) Cn extract alone and (F) Chloramphenicol. **b** Antibacterial activity of Cn-AgNPs against *E. coli* when treated with (A) Cn extract alone and Cn-AgNPs of (B) 25 µg/mL, (C) 50 µg/mL, (D) 75 µg/mL, (E) 100 µg/mL and (F) Distilled water. **c** Antibacterial activity represented as bar plot. **d** Resazurin assay for determining the minimum inhibitory concentration of Cn-AgNPs against *S. aureus & E. coli*. Serial dilutions were made from the first well (100 µg/mL to 0.39 µg/mL). Tenth and eleventh well represents the negative and positive control respectively. **e** Comparison of DPPH radical scavenging activity of Cn-AgNPs, Cn aqueous extract, Cn-AgNP+ Ascorbic acid and Ascorbic acid (AA)

The comparison of DPPH radical scavenging activity of Cn-AgNPs, Cn aqueous extract, Cn-AgNP + Ascorbic acid and Ascorbic acid (AA) is shown in Fig. 7e. The antioxidant moieties reduced the purple coloured DPPH free radical and the colour intensity got decreased was measured at 517 nm. The percentage of DPPH scavenging activity shows a concentration dependant increase from 25 to 100 μg/mL of Cn-AgNPs, with an IC_50_ of 57.8 μg/mL. IC_**50**_ of Cn aqueous extract, Cn-AgNP+ Ascorbic acid and Ascorbic acid (AA) are 92.7, 26.4 and 35.87 μg/mL respectively. The significant antioxidant activity of Cn-AgNPs which is comparable to ascorbic acid might be due to a bioactive capping agents on their surface. When combined with Cn-AgNPs, ascorbic acid can enhance the stability and activity of AgNPs by preventing their oxidation and aggregation. Both Cn-AgNPs and ascorbic acid can scavenge reactive oxygen species (ROS) effectively. When used together, they can complement each other's antioxidant activities, leading to a more efficient neutralization of ROS. This synergy results in a higher overall antioxidant capacity compared to using either compound alone. Silver nanoparticles can facilitate the regeneration of ascorbic acid from its oxidized form, dehydroascorbic acid (DHA). Owing to this regeneration process, ascorbic acid retains the antioxidant activity for a longer period of time in the presence of Cn-AgNPs. Cellular absorption of AgNPs can be improved by ascorbic acid. Once inside the cells, these substances can complement one another to neutralize ROS and strengthen antioxidant defences within the cells, thereby preventing oxidative damage [77]. Excellent antioxidant activity exhibited by flavonoids and phenolic chemicals enables them to be employed to prevent and treat degenerative illnesses [78]. Therefore, the increased antiradical activity comparable to that of the control ascorbic acid is mostly initiated by the bioactive chemicals that are present on the surface of green synthesised nanoparticles. However, Cn-AgNPs' distinct size-dependent characteristics might be the cause of its increased antioxidant activity when compared to Cn extract. By stabilizing the plant extract's bioactive components, nanoparticles can improve its antioxidant qualities. However, the upsurge in the antioxidant potential of Cn-AgNPs compared to Cn extract may be due to its unique size dependent properties. Nanoparticles can stabilize the bioactive compounds in the plant extract and can thus enhance the antioxidant properties. Reactive oxygen species primarily alter the redox state of cell membrane proteins, increasing the rates of melanin synthesis and resulting in dark skin [79]. Hence the antioxidant activity of Cn-AgNPs boosts its applicability as a cosmetic agent, since this effect can augment skin rejuvenation as well.

### Antibacterial studies

#### Agar well diffusion method

The agar well diffusion method was utilized to assess the antibacterial efficacy of Cn-AgNPs that were synthesized by the green approach. For the analysis, ATCC 25922 and ATCC 25923 cultures were utilized. The Cn-AgNPs (100 µg/mL) demonstrated excellent antibacterial efficiency against gram positive (Staphylococcus aureus) and gram negative (Escherichia coli) pathogenic bacteria, as confirmed by the diameters measured for the zone of inhibition (Table 2). This performance was comparable to that of the positive control, chloramphenicol. When compared to AgNPs, plant extract by itself was unable to demonstrate any antibacterial activity, suggesting that the silver nanoparticles were the only source of the antibacterial effect (Fig. 7a–c). The mode of action of AgNPs against bacteria is still not well understood. Silver ions released from AgNPs are readily able to enter the cell, which may alter membrane permeability and denaturize proteins by interacting with sulfhydryl groups, ultimately causing bacterial mortality. There have also been studies confirming the intracellular leakage of ATP by the generated silver ions. Moreover, cell death may arise from cytoplasmic fluid leakage followed by cell shrinkage [80]. According to Gupta et al., catalase inhibition, free radical-induced membrane damage, and free radical production are important factors in cell death [81]. Several published studies examining the antibacterial properties of silver nanoparticles against both gram-positive and gram-negative bacteria revealed that the antibacterial activity of silver nanoparticles was somewhat greater against gram-positive bacteria [82, 83]. Because of structural variations in their cell walls, gram-negative bacteria are typically more resistant to silver nanoparticles than gram-positive bacteria. Lipopolysaccharides (LPS) make up the outer membrane of gram-negative bacteria, which serves as a barrier to keep out antimicrobial treatments like silver nanoparticles. The outer membrane acts as an extra line of defence, obstructing the penetration of silver nanoparticles and preventing them from exerting their antibacterial impact [84]. Moreover, gram-negative bacteria have efflux pumps, which are specialized proteins that actively remove harmful materials from the bacterial cell, including silver nanoparticles. By assisting the bacterium in eliminating silver nanoparticles and lowering their intracellular concentration, this efflux mechanism strengthens the organism's resistance. Gram-positive bacteria, on the other hand, have a thicker layer of peptidoglycan in their cell walls and no outer membrane. Because there are fewer barriers preventing the silver nanoparticles from penetrating and interfering with cellular processes, their comparatively simpler cell wall structure renders them more vulnerable to the antibacterial activity of the particles [85]. Gram-negative bacteria are more resistant to silver nanoparticles than gram-positive bacteria because of the combined action of their efflux pumps and outer membrane. Furthermore, the bacterial cell wall's surface charge influences the way bacteria interact with nanoparticles. The cell wall surface of gram-positive bacteria is usually more positively charged, which makes it easier for negatively charged silver nanoparticles to adhere to them and increases their antibacterial activity.

**Table 2:** Zone of inhibition, MIC and MBC of Cn-AgNPs (µg/mL) on *E. coli* and *S. aureus*

Bacterial strains	Cn-AgNPs concentration (µg/mL)		
	MIC	MBC	Inhibition zone (mm)
*Escherichia coli* (ATCC 922)	6.25 µg/mL	7. 9 µg/mL	12
*Staphylococcus aureus* (ATCC 923)	3.125 µg/mL	3.7 µg/mL	14

### Determination of MIC and MBC

Cn-AgNPs evidenced to be effective against gram-positive and gram-negative bacteria in terms of antibacterial activity. The metabolization of resazurin was used to assess the effectiveness of Cn-AgNPs as an antibacterial agent. When there is no bacterial growth, the resazurin indicator is blue/purple; when growth is present, it is pink or colorless (Fig. 7d). The MIC and MBC values of Cn-AgNPs against S. aureus and E. Coli are recorded in Table 2. The greatest antibacterial effectiveness of Cn-AgNPs was demonstrated at the lowest concentration of 6.25 µg/mL against E. Coli and 3.125 µg/mL against S. aureus. Reports suggest that the bactericidal effect may be caused by Ag + interacting with the negatively charged bacterial cell membrane. Nanosized particles can enter the cell membrane more quickly due to increased cell permeability and positive surface density, which in turn results in cell death. [86]. The minimal bactericidal concentration was attained and, upon plating the contents of the first four wells, no discernible growth was seen after the 24-h incubation period. These findings, however, point to the potential use of Cn-AgNPs as an antibacterial agent. One common infectious agent on skin is S. aureus. Therefore, in addition to its cosmetic benefits, Cn-AgNPs' antibacterial action helps shield against skin infections.

## Conclusion

In this study the antioxidant, antibacterial, antityrosinase and antimelanogenesis activity of the green synthesised silver nanoparticles were studied. We established a multifunctional silver nanoparticle which could act as skin lightening agent synthesised by the reduction caused by the leaf sheath scales of *Cocos nucifera* aqueous extract. The bioactive components of the extract cause the reduction of Ag^+^ to Ag^0^. UV–Visible spectroscopic results confirmed the formation of silver nanoparticles. FTIR results confirm the presence of active biomolecules responsible. It is noteworthy that Cn-AgNPs efficient activity against Tyrosinase enzyme proclaimed its infinite possibilities in research fields especially in cosmetics and pharmaceuticals. It is promising that these synthesised silver nanoparticles are of strong antibacterial agents against the causative microbes of skin infections and their antioxidant properties can enhance skin rejuvenation. Thus, the findings presented in the study suggests an environmentally friendly material that could be used as a cosmetic formulation. Further preclinical in vivo studies in this direction can aid the development of an effective cosmetic product, using Cn-AgNPs.

## Data Availability

The data used to support the findings of this study are available from the corresponding author upon request.
